# Adjunctive techniques for renal cell carcinoma ablation: an update

**DOI:** 10.3389/fradi.2025.1559411

**Published:** 2025-03-17

**Authors:** Tiago Paulino Torres, Ioanis Liakopoulos, Vasilios Balomenos, Stavros Grigoriadis, Olympia Papakonstantinou, Nikolaos Kelekis, Dimitrios Filippiadis

**Affiliations:** ^1^ Interventional Radiology Department, Hospital Trás-os-Montes and Alto Douro, Vila Real, Portugal; ^2^ Interventional Radiology Department, 251 General Aviation Hospital, Athens, Greece; ^3^ Interventional Radiology Department, University General Hospital Attikon, Athens, Greece

**Keywords:** renal cell cancer, adjunctive techniques, embolization, thermal ablation, cryoablation

## Abstract

Percutaneous ablation therapies currently play a major role in the management of T1a and T1b renal cell carcinoma (RCC). These therapies include thermal ablative technologies like radiofrequency (RFA), microwave (MWA) and cryoablation, as well as emerging techniques like irreversible electroporation (IRE) and high-intensity focused ultrasound (HIFU). These therapies are safe and effective, with their low complication rate being mostly related to the minimal invasive character. To increase the outcomes and safety of ablation, particularly in the setting of larger tumors, adjunctive techniques may be useful. These include pre-ablation trans-arterial embolization (TAE) and thermal protective measures. TAE is an endovascular procedure consisting of vascular access, catheterization and embolization of renal vessels supplying target tumor, with different embolic materials available. The purpose of combining TAE and ablation is manifold: to reduce vascularization and improve local tumor control, to reduce complications (including the risk of bleeding), to enhance tumor visibility and localization, as well as to improve cost-efficiency of the procedure. Thermal protective strategies are important to minimize damage to adjacent structures, requiring accurate knowledge of anatomy and proper patient positioning. In RCC ablation, strategies are needed to protect the adjacent nerves, as well as the visceral and muscular organs. These include placement of thermocouples, hydro- or gas-dissection, balloon interposition, pyeloperfusion and skin protection maneuvers. The purpose of this review article is to discuss the updated role of ablation in RCC management, to describe the status of adjunctive techniques for RCC ablation; in addition it will offer a review of the literature on adjunctive techniques for RCC ablation. and report upon future directions.

## 1 Introduction

Renal cell carcinoma (RCC) represents about 3% of all forms of cancer and about 90% of malignant tumors of the kidney (1). The three primary types of RCC are clear cell, papillary, and chromophobe (constituting up to 70%–85%, 10%–15%, and 4%–5% of cases, respectively). A minority of solid kidney neoplasms, ranging around 2.5%–18%, is cystic renal cell carcinoma. The occurrence of this cancer, including all stages, has been increasing over several years, leading to steadily increasing mortality rates per population unit (2–4). RCC is a male-predominant (with the ratio being 2 to 1) disease with a typical presentation in the sixth and seventh decades of life and median age being about 60 years (2). An established risk factor for renal carcinoma are active and passive cigarette smoking, with a relative risk of about 2 to 3 (5, 6). Obesity, and more particularly BMI (body-mass index), is a known risk factor as well (7, 8). Hypertension is another established risk factor, with data suggesting that antihypertensive medications such as diuretic drugs are not independently associated with development of this cancer (9). RCC seems to be more common in patients with end-stage renal failure, acquired renal cystic disease, and tuberous sclerosis than in the general population (10, 11). For most patients, no identifiable risk factor can be determined, and the mechanisms by which pathogens interact with known risk factors remain unclear at present. About 2%–3% of cases are familial and several autosomal dominant syndromes are described, each with a distinct genetic basis and phenotype, with von Hippel-Lindau syndrome to be the most notable of all (12). Von Hippel-Lindau patients inherit a defect on one allele of the VHL gene and a defect in the other allele is acquired in affected organs. Renal cell tumors in this syndrome thus tend to be of early onset and multifocal. Most patients with sporadic (non-inherited) clear cell renal tumors acquire defects in both VHL alleles, resulting in dysfunction of the von Hippel-Lindau protein. Sporadic clear cell cancer thus tends to be late onset and unifocal.

Patients may present with either local or systemic symptoms. However, most of the presentations are identical, given the widespread use of abdominal imaging. Local signs and symptoms include haematuria, flank pain, or a palpable abdominal mass, all of which have negative prognostic implications. Systemic symptoms can be caused from metastases or paraneoplastic events, largely related to secreted proteins, such as parathyroidhormone-related protein (causing hypercalcaemia), renin (causing hypertension), erythyropoietin (causing erythrocytosis) and fever.

For localized disease, the 5-year survival rate for kidney cancer is 91.8%, while for advanced disease the equivalent rate is 12.1%. The most prevalent prognostic factors are the tumor grade, the presence of nodal or distal metastases at presentation and the local extent (13). In regards to metastasis, the most common reported sites include the lungs, brain, bone, adrenal glands and liver. Prevalent use of ultrasonography and cross-sectional imaging is nowdays associated with incidental detection of many asymptomatic kidney tumors. As a result, since the increased detection of incidental renal masses is directly related to reduction in presentation of synchronous metastatic disease, this cancer is often detected at quite early stages. Tumor staging is accomplished mainly with CT, which allows for assessment of local invasiveness, lymph node involvement or other metastases. TNM system is currently used for the staging of renal cell carcinoma;T1 stage is currently an indication for percutaneous ablation. According to the TNM staging system T1 stage refers to a tumor of a diameter up to 7 cm (T1a up to 4 cm and T1b 4.1–7 cm) confined to kidney with no spread to nearby lymph nodes or distant organs.

While partial nephrectomy is considered the gold-standard for the management of localized RCC, recently ablative therapies are emerging as equivalent alternatives with comparable rates, oncologic outcomes and fewer complications (including minimum or no impact to renal function). Thermal ablation, in the form of radiofrequency ablation, microwave ablation and cryoablation, is being increasingly accepted by scientific societies and multidisciplinary international guidelines and is particularly recommended in patients with significant renal impairment, old age, comorbidity burden, renal impairment, old age or in patients unwilling to undergo surgery. The above-mentioned management strategies are used more confidently and systemically in all patients, since the maturation of long-term oncologic outcomes. Aiming to increase the outcomes and safety of ablation, particularly in the setting of larger tumors, adjunctive techniques can be useful.

The purpose of this review article is to discuss the updated role of ablation in RCC management, to describe the status of adjunctive techniques for RCC ablation; in addition it will offer a review of the literature on adjunctive techniques for RCC ablation. and report upon future directions. This is not a systematic review of the literature. A number of separate literature searches were performed. Non-English studies and case reports were excluded from the study. All references of the obtained articles were also evaluated for any additional information.

## 2 Management of RCC (stage I)

RCC treatment strategy varies according to the tumor stage. The treatment options according to the current NCCN guidelines for T1a tumors include partial nephrectomy as the preferred option and ablation therapies or active surveillance as alternatives (14). They also include radical nephrectomy in select patients. For T1b tumors the guidelines include the ablative therapies as an option in well selected patients, including non-surgical candidates, patients with co-morbidities or those refusing surgical options (14).

### 2.1 Surgical therapy

There are two surgical approaches for the treatment of RCC Stage I, partial nephrectomy (PN) and radical nephrectomy (RN). PN has shown comparable oncologic outcomes data to RN (15, 16). PN is a minimally invasive surgical technique with maintenance of renal function. On the contrary, RN reduces renal function (especially in patients with impaired renal function) which is a significant prognostic factor for morbidity and mortality (17). PN is most appropriate when preservation of renal function is a primary issue, such as in patients having one kidney or those with renal insufficiency, bilateral renal masses or familial RCC. Partial nephrectomy is also appropriate for patients at relative risk of developing progressive chronic kidney disease due to young age or medical risk factors (e.g., hypertension, diabetes, nephrolithiasis). Feasibility of the resection depends on the anatomical characteristics of the lesion; lesions in the poles of the kidney are more suitable for PN than lesions in proximity to the renal pelvis. In order to classify objectively the anatomical characteristics of the renal masses and to plan surgical resection, specifically described nephrometry scoring systems have been introduced and incorporated into clinical practice (13, 18, 19). In case of PN, both open and laparoscopic approaches can be considered, depending on tumor size, location and the surgeon's expertise. According to the results of a population based analysis 10% of patients undergoing partial nepherectomy have intra-operative conversion to radical nephrectomy (20).

### 2.2 Active surveillance (AS)

The majority of small renal tumors have a slow growth pattern (about 3 mm per year) (21). So one approach would be to follow up them with cross-sectional imaging in tactical basis, in order to check their size over time. On the contrary, it is reported that there is a number of small renal tumors with aggressive characteristics (21). In addition to that, it has been found that renal tumors less than 4 cm is possible to have nodal or distant metastases (22, 23). Key factors of choosing active surveillance for the management of a renal lesion are the age of the patient, his performance status and the size of the lesion (13, 23). The multidisciplinary tumor board (MDT) of each facility has the responsibility to make the election of the patients which are suitable for this type of treatment. According to the NCCN guidelines, AS is an option for the management of localized small renal masses (under 3 cm) and should be a primary consideration for patients with decreased life expectancy or extensive comorbidities that would be at excessive risk for more invasive intervention (14).In a Medicare linked population retrospective propensity score-matched study of patients with T1aN0M0 RCC cancer-specific as well as overall survival for all active surveillance comparisons were significantly lower (24).

### 2.3 Ablation therapy (AT)

Ablative therapies include radiofrequency ablation (RFA), microwave ablation (MWA) and cryoablation (CA). They are minimally invasive therapies, utilising heat- or cold-based energy, in order to necrotize the tumor. Literature data provide evidence upon safety, efficacy, low cost of ablative therapies with similar to surgical approaches and oncologic outcomes; compared to surgical options ablative therapies have been favoured for a significantly better preservation of the overall kidney function and lower complication rates (25–28). In a systematic review and meta-analysis including 38 studies with >3,000 patients, percutaneous cryoablation for stage I RCC (either T1a or T1b) was found to have minimal significant impact on renal function (measured by eGFR or serum creatinine); the same conclusion was true for patients with solitary kidneys as well (29). In a 10 year prospective study and comparison with matched cohorts from the National Cancer Database percutaneous cryoablation yielded a 10-year disease-specific survival of 94%, which was equivalent to that reported after radical or partial nephrectomy; furthermore the overall survival probability after percutaneous cryoablation at 5 years and 10 years was longer than for radical or partial nephrectomy, especially for patients at higher risk (Charlson/Deyo Combined Comorbidity score ≥2) (30).

## 3 Adjunctive techniques for RCC ablation

### 3.1 Trans-arterial embolization (TAE)

Ever since the first description of TAE being applied as an ancillary treatment for RCC prior to percutaneous ablation, it has been increasingly recognized as a safe and effective procedure, particularly helpful in the context of larger tumors (31–34); however, there's still a lack of prospective trials and large cohorts regarding combined therapy of TAE+ablation of RCC in the available literature. This combined technique is included in the Cardiovascular and Interventional Radiological Society of Europe (CIRSE) Standards of Practice document and the Society of Interventional Radiology (SIR) Quality Improvement Standards document regarding percutaneous ablation in RCC (13, 35).

Regarding the technical aspects of TAE procedure, this usually includes arterial vascular access typically via the right common femoral artery (radial access is also possible) under ultrasound guidance, with a vascular sheath (most commonly 5 Fr) being placed. Selection of the renal arteries can be performed with shaped catheters of choice, with or without previous flush catheter aortography to identify the origin of the renal arteries. Once the renal artery is selected, angiography is performed to identify the tumor, assess its vascularity, and identify the target feeding vessels supplying the tumor. Super-selective catheterization of these vessels can be performed with a microcatheter and microwire. Embolization materials can then be deployed, with or without occlusion balloon catheter (32, 36).

There's a large heterogeneity of chosen embolic agent(s) for pre-ablation TAE in RCC. The earliest reports of renal embolization for RCC in the English literature date back to 1971 including case series of TAE performed with autologous muscle particles (from quadriceps femoris muscle group) (37, 38). Since then, different materials have been used in TAE, including both non-permanent and permanent embolic materials (39). Non-permanent materials include resorbable gelatin sponge, iodized oil (Lipiodol) and degradable starch microspheres. Permanent embolic materials include liquid agents like ethanol; medical glue: n-butyl cyanoacrylate (Histoacryl), n-butyl cyanoacrylate and metacryloxisulfolane (Glubran 2); and ethylene vinyl alcohol (EVA)-based liquid material (Onyx) dissolved in dimethyl sulfoxide (DMSO). Permanent embolic materials also include solid particles, which can be with or without spherical shape, size-calibration and/or drug-elution. Non-tightly-size-calibrated particles include polyvinyl alcohol (PVA), tris-acryl gelatin microspheres and sodium acrylate alcohol co-polymer. Tightly-size-calibrated particles include polyzene-F coated hydrogel and polyethylen glycol components. Coils and micro-coils can also be used.

There is no defined standardized protocol of choice and preparation of the materials (e.g., dilution ratio of gelatin sponge with non-ionic iodinated contrast media; water-in-oil emulsion ratio if combining Lipiodol with microspheres; Lipiodol:glue ratio; particle size) about RCC TAE (36, 39). Regardless of the embolic agent, TAE is performed until stasis is achieved in the target tumor feeding vessels (31, 36). But in this context, there is no relevant data to affirm that one embolic choice, or a specific combination is superior to the other (31, 40). Since renal arteries are considered “end vessels” and since RCC is considered a hypervascular tumor, a desired approach would include an embolic agent that at least results in permanent small vessel occlusion (36); in addition, large vessel embolization with coils may also be warranted (36).

Regarding the time between TAE and ablation**,** there's a lot of heterogeneity in the literature, with intervals of few minutes/hours up to a day or several days (41–43). There is still no data on what the interval between these procedures should be and if this affects outcome. However, a short time interval—ideally a single session treatment—may be desirable from both the patient and logistical standpoint, though prospective data with outcome evaluation and cost analysis is warranted. Larger cohorts, prospective and comparative data are necessary to establish a preferred embolic choice and technique protocol for pre-ablation TAE in RCC treatment.

**Figure 1:**
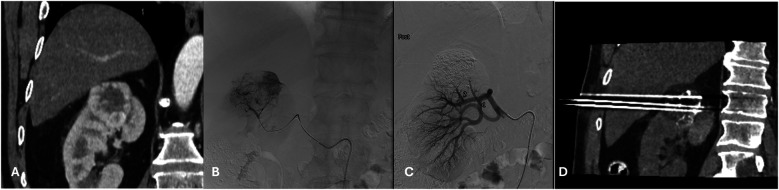
**(A)** A 71 years-old male patient with a congenital solitary kidney and a biopsy proven T1b RCC in the upper renal pole. **(B)** Fluoroscopy image prior to embolization with lipiodol and microspheres. **(C)** Fluoroscopy image post embolization with lipiodol and microspheres. **(D)** The morning post TAE percutaneoys cryoablation was performed with a transhepatic approach and placement of 3 cryoprobes.

The rationale for pre-ablative embolization of the arteries supplying the RCC is manifold (Figure 1). TAE as an adjunctive technique has the potential role of improving local renal tumor control (44); the rationale for this approach includes reducing the tumor vascularity, effectively reducing the arterial heat- (or cold-) sink effect while combining thermal and ischemic tumor necrosis (39). Some cohort studies combining TAE + RFA report local tumor control rates of 97%–100% (36). Mahnken et al. reports one of the earliest experiences combining TAE + RFA (within 24 h between procedures) (42); in this cohort, 6 tumors with >3 cm (not exceeding 4 cm) were treated with this combined therapy, showing complete ablation, no local recurrence and no major complications, with a mean follow-up period of 13.9 ± 12.4 months. In 2007, Arima et al. (43) published the largest case series to date, reporting the results of TAE + RFA of 36 RCC tumors (mean diameter 3.1 cm; ranging from 1.2 to 6.5 cm) in 31 patients. TAE was performed with ethanol mixed with either iodized oil or polyvinyl alcohol, less than a week prior to RFA procedure. In this study, no recurrence was noted in RCC < 4 cm cases during a mean follow-up period of around two years, with a recurrence rate of 2.8% in tumors larger than 4 cm. Nakasone et al. reports a cohort of 10 patients with 12 RCC tumors (mean size of 3.1 cm, ranging from 1.8 to 6.6 cm), submitted to a single session of combined TAE (gelatin sponge and iodized oil) + RFA (few hours between both procedures) (41). No local tumor was identiﬁed on a mean follow-up of around 47 months (41). There was no significant effect on patient glomerular filtration rate (GFR) and serum creatinine levels. No major complications were reported; however, half of the patients experienced back pain after the procedure. Michimoto et al. (45) retrospectively evaluated the use of combined TAE and cryoablation in a cohort of 17 patients with endophytic renal masses (mean size of 2.65 cm). The authors performed TAE using a mix of absolute ethanol and iodized oil to improve identification of the renal masses on unenhanced CT scans prior to CT-guided percutaneous cryoablation. TAE was successful in 16 out of 17 patients. Cryoablation was successful in all patients with local tumor control rate of 93% at a mean follow-up of 15.4 months. This study, on the other hand, revealed a statistically significant drop in estimated GFR after procedure.

Historically, most of these studies focus on the combined treatment of renal lesions with a mean diameter <4 cm, that is, mostly T1a RCC tumors (36). However, some studies have also been showing promising results in patients with larger mean tumor diameters, where pre-ablation TAE can potentially play a more important role in helping achieve complete tumor control and enhance percutaneous ablation (33, 36). In fact, given the recent inclusion of ablative techniques as a treatment for T1b RCC in select patients (as per NCCN guidelines), these combined therapies can be of utmost importance in the treatment of larger renal tumors from now on (14).

Yamakado et al. reported the combined therapy of TAE + RFA (using either ethanol and iodized oil mixture or PVA particles) in a small cohort of 11 patients (total of 12 RCCs treated) with mean RCC diameter of >5 cm (ranging from 3.5–9 cm), with all tumors showing significant size reduction, complete eradication of tumor enhancement one week following ablation, and successful local control over a 13 month period (46). A study by Duan et al. (47) describes a series of 28 patients with average tumor size of 6.7 ± 2.2 cm (ranging from 4.1–9.6 cm) treated with TAE+RFA (5–7 days between procedures). TAE was performed with iodized oil and gelatin sponge particles. The procedure was technically successful in all patients; tumor enhancement disappeared after a single session in 20 patients, after 2 sessions in 4 patients and after 3 RAE-RFA sessions in the other 4 patients. 2 of the patients died of other causes, and in the remaining 26 patients, tumors remained controlled during a mean follow-up period of 27 months with significant reduction in size (from (6.7 ± 2.2 cm to 3.9 ± 1.7 cm), without significant changes in creatinine levels nor serious complications.

As for TAE+cryoablation in the treatment of larger lesions, Gunn et al. retrospectively review 9 patients (mean tumor diameter of 5.17 cm) showing that this combination therapy is safe and technically feasible (40). In this study, TAE was performed with a wide variety of operator-dependent choice and combination of various embolic materials, including particles (size ranging from 250 to 900 µm) mixed with contrast and/or iodized oil. This study demonstrated that TAE+cryoablation did not result in adverse outcomes or increased complications but also showed that no improvement of technical success or clinical outcomes could be identified by propensity score matching analysis (the 9 patients who underwent combination therapy were matched in a 2:1 ratio with patients who underwent cryoablation alone, using age, gender, and tumor size) (40). Li et al. (48) describes a larger group of 32 patients with RCC submitted to TAE and cryoablation (2–3 weeks apart); with average tumor size of 9.8 ± 3.4 cm (ranging from 4.0 cm to 19.8 cm) and tumor necrosis rate of 57.5 ± 17.51% at a month follow-up.

MWA therapies usually create larger ablation areas compared to other heat ablation techniques, thus can potentially be useful in treatment of larger renal lesions (32, 49). However, the literature on combining TAE+microwave ablation (MWA) is scarce; a scientific poster (44) reports a retrospective review of 11 patients who underwent combined single-day TAE+MWA, with average tumor size was 4.5 cm (9 out of 11 tumors were clear cell carcinoma, one papillary carcinoma, and one oncotic neoplasm). Technical success was achieved in all cases with no recurrence on an average follow-up of 297 days. This report identifies TAE + MWA as a safe and effective technique in treating large renal tumors, with large prospective trials being needed for further validation.

TAE presents the potential benefit of reducing the risk of bleeding during ablation procedures (31, 39, 50). This can be useful before heat ablation (34) or cryoablation of larger tumors (51, 52). Particularly in the latter setting, partly due to the intrinsic fact that surrounding vessels are not directly cauterized with cryoablation; there might also be a need for multiple cryoprobe placement, a need for central renal probe placement, or the risk of ice ball cracking—all factors that can lead to significant bleeding (52).

However, the available data to support this potential benefit is currently conflicting; a retrospective review by Woodrum et al. demonstrated 10 patients who underwent percutaneous cryoablation of large renal tumors (>5 cm) (4 of them underwent TAE+cryoablation, with microspheres or particles; 6 patients underwent cryoablation alone; without significant differences in the tumor size, number of probes used or renal function between these two groups) (51). Cryoablation was successfully performed in all 10 patients. The mean post-ablation hematoma volume in patients who underwent TAE+cryoablation was significantly lower than those who underwent cryoablation alone, hinting at some protective effect of combination therapy against post-procedural bleeding.

In contrast, the previously mentioned recent retrospective review by Gunn et al. (40) showed no statistically significant differences in post-procedural hematocrit value drop (as a surrogate for bleeding) when comparing combination therapy and cryoablation alone; alas, no other objective benefits (improvement of technical success or reducing complication) over cryoablation alone could be identified by propensity score matching analysis (40). Similarly, a recent retrospective study comparing the outcomes of TAE and cryoablation vs. cryoablation alone in patients with T1b and T2 RCC tumors showed that the mean volume of post-ablation hematomas in the combined treatment group was less than half than those treated with cryoablation alone, although this did not reach statistical significance (53). Larger cohorts and prospective studies are needed to properly evaluate the added value of combination therapy to potentially decrease the risk of post-procedural bleeding.

As previously stated, RCC ablation procedures might need the use of multiple probes, particularly (but not only) in the setting of cryoablation (39, 54). Pre-ablation TAE of renal tumors, when routinely included in ablation protocols, can help reduce tumor volume and therefore, greatly reduce the number of necessary probes (54, 55) when performing the ablation, even by half as described in a technical report (54); this, in turn, can decrease the total procedural cost (even by 15%) (27) and improve its cost efficiency.

TAE may help to enhance tumor localization during ablation, by tagging the lesion with different embolic materials; the prime example of this application is ethiodized oil: its retention within the tumor helps to localize RCC during non-enhanced CT-guided percutaneous ablation, which may be particularly helpful in some scenarios like multiple masses in the same kidney or endophytic location (32, 45). This also allows for a better depiction of the tumor edge as well as a safe margin for ablation (45).

One promising alternative to iodized oil are visible beads. This option can be advantageous for simultaneous tumor demarcation and ischemic tumor necrosis (39). An example of this includes calibrated, radiopaque, biocompatible, non-resorbable hydrogel beads (70–150 μm) which have been used in trans-arterial chemoembolization (TACE) of hepatocellular carcinoma (HCC) in the last few years (56). Their use in TAE/TACE of renal tumors is still to be reported in the English literature, as of now. Other materials have been used to create visible beads, but *in-vivo* clinical data regarding safety and effectiveness are still lacking in the literature (39).

Complications from this combined procedure can be broadly divided into ablation-related and TAE-related complications. Overall, RCC thermal ablation is considered a safe procedure, with major complications occurring in a minority of patients (most commonly: hemorrhage, abscess, unintentional damage to adjacent structures such as ureter, bowel, genitofemoral nerve, psoas muscle) and tract seeding (34, 35). Post-ablation syndrome is a self-limiting condition occurring in less than 10% of patients and should not be considered as a complication but as an expected/accepted side effect (35). In the literature, rates for specific types of adverse events are largely dependent on patient selection and are mostly based on case series consisting of several hundred patients (34). That being said, a review including 254 RFA procedures (mean size of RCC tumor: 2.1 ± 0.8 cm) reports an overall complication rate of 9.8% and major complication (considered Clavien–Dindo grade II–IV) rate of 4.7% (30), with the most common complications being urothelial stricture (2.1%) and nerve injury (3.9%) (52, 57). Other complications included vascular injury, urine leakage, infection, pneumothorax and medical events (atrial fibrillation, hypertension, supraventricular tachycardia) (57), among others. The literature on MWA experience is scarcer, but a retrospective study of 106 patients with mean RCC diameter of 2.4 ± 0.7 cm revealed an overall complication rate of 5.7% (58), which included five small perinephric hematomas (Clavien–Dindo grade I) and two pneumothoraxes (Clavien–Dindo grade III). Due to its larger potential ablation zone, MWA has been used in larger T1b tumors: the literature describes complication rates of 3%–17%, including perirenal hematoma, urinoma formation, and skin dysesthesia( (26, 52). Overall complications following MWA of larger renal masses appear to be like the treatment of T1a tumors (52), although further prospective studies are needed.

Regarding cryoablation, a review of 311 cryoablation procedures (mean size of RCC tumor: 3.2 ± 1.3 cm) describes an overall complication rate of 13.2% with a major complication (Clavien–Dindo grade II–IV) rate of 8.4% (30). The most common complications were hemorrhage/vascular injury (4.8%) and hematuria (2.6%). Of note, a more recent single-center, retrospective study evaluating the long-term outcomes of 54 cryoablated RCCs, mostly T1a (49/54 lesions), reported no complications of grade III or greater, with grade II complication rate of 7.8% (59).

Of note, most of the literature reporting thermal ablation for T1b RCCs describes the use of cryoablation (32), for multiple reasons (operator choice, ability to use multiple probes, direct visualization of ice-ball/ablation zone under CT guidance). The described overall rate of major complications after cryoablation for T1b RCCs goes up to 16.2% (3, 34) in a series including 37 treated patients (mean RCC size of 4.7 ± 0.63 cm) (60); these adverse events include hemorrhage, abscess/infection, bowel injury, pneumothorax, medical renal failure, urinary collecting system injury, or nerve injury (32, 35). As of now, the reported literature does not provide enough data to separate out rates of individual complications in the context of T1b RCC ablation (32, 35).

When comparing complications derived from heat-based techniques and from cryoablation, recent reviews found no statistically significant difference in the rate of major complications; even though cryoablation showed a higher incidence of peri-renal hematomas (61).

Regarding TAE-related complications, these are usually rare, operator-dependent, and may be due to issues with suboptimal arterial access (pseudoaneurysm in the site of puncture; retroperitoneal hematoma), vessel dissection during catheterization and off-target embolization. Recent case series do not report any serious TAE-related complication in this context (62), without any prospective studies available in the current literature.

### 3.2 Thermal protection

Percutaneous image-guided thermal ablation procedures are considered safe and effective, with low complication rates usually arising from bleeding and unintentional damage to adjacent structures (34, 35, 63). Therefore, careful treatment planning is warranted for a successful procedure and to avoid complications (34, 64), including choosing the best ablation technology and imaging technique, patient positioning, anatomical considerations and anesthesia (34, 64). On that note, some useful algorithms to plan a renal ablation procedure include the RENAL nephrometry scoring system (to better stratify the renal lesion according to its complexity) (65) and the ABLATE approach (66).

One should consider the surrounding critical anatomical structures that might be damaged during ablation (64, 66, 67). Some of the adjacent major organs at risk during renal ablation include: small bowel and colon (risk of perforation/fistula, bleeding and infection), liver and spleen (main concerns include bleeding and biliary damage), adrenal glands (possible acute hypertensive crisis), pancreas (risk of fistula and pancreatitis), the collecting system itself (ureter and pelvi-ureteric junction) (risk of fistula/urinoma or stenosis) and muscles (namely, psoas muscle) (68). Surrounding nerve structures should also be considered. Due to their topography, relevant nerves at risk during renal ablation include the lumbar plexus and its sensitive and motor branches (67). The lumbar plexus (formed by the ventral rami extending from L1–L5) is located posteromedial to and within the posterior portion of the psoas muscle (67). Its primary sensory nerves include the iliohypogastric (T12–L1; innervation of hypogastric region), ilioinguinal (L1; innervation of inguinal region, medial thigh and scrotum/labia), genitofemoral (L1–L2; innervation of femoral triangle, anterior thigh and scrotum/labia) and lateral femoral cutaneous (L2–L3; innervation of anterolateral thigh) nerves (67). The femoral nerve (origin from L2–L4 rami of lumbar plexus) is also at risk, and it provides both sensory (anteromedial thigh and medial knee and leg) and motor supply (iliacus, pectineus, and quadriceps muscles) (67). Thus, nerve injury from thermal ablation in these locations may provoke sensory (anesthesia, paresthesia or dysesthesia) or motor (paralysis or paresis) deficits (67). These injuries may be temporary or permanent, depending on the duration of the injury and the achieved temperature (67). A safe temperature limit to avoid nerve injury is between 10 and 44°C (67). Thermocouples or fiberoptic thermosensors and electromyography (for motor-evoked potential monitoring) can be used during ablation procedures to monitor local temperature and nerve function, respectively, to avoid complications (67).

The literature reports an ideal minimum safe distance of 1 cm between the ablation zone and relevant structures to avoid complications (63, 66, 67). The following different techniques can be used to achieve this distance and/or protect the anatomical structures during renal ablation.

As previously stated, proper patient positioning is an essential part of planning; positioning (possibly aided by vacuum mattress or external compression) may be enough to displace surrounding structures and achieve proper thermal protection (4); this should be taken into account before further deciding to use more adjunctive techniques.

**Figure 2:**
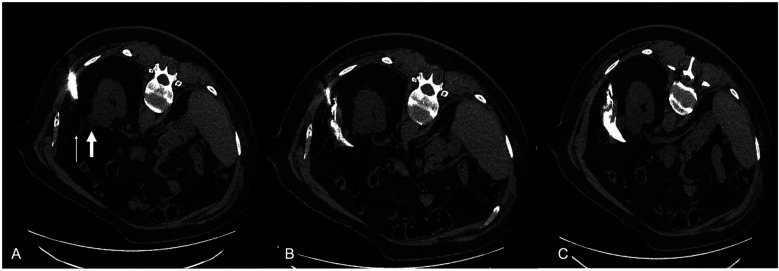
**(A)** A 68 years-old male patient with a biopsy proven T1a RCC in the upper renal pole of the left kidney (thick white arrow) in close proximity to the large intestine (thin white arrow). **(B,C)** A 15 Gauge dissection needle with spring loaded blunt tip stylet was placed between the tumor and the intestine and normal saline mixed with contrast medium was injected to increase the distance between the two.

Hydrodissection consists of fluid injection directed to the spaces surrounding a target lesion, to displace and thermally insulate the adjacent organs (63, 68). This can be done using low caliber needles (e.g., 21–22G spinal needle) under image-guidance. Injected fluid can be composed of pure 0.9% saline (NaCl) solution, while some authors prefer to use a 2%–5% solution of iodinated contrast diluted in saline (41) for better visualization under CT. Imaging is usually acquired after injection of fluid to confirm its position (Figure 2). The needle can be left in place for subsequent instillation during the procedure (67). Continuous injection of small amounts of ﬂuid can also be used to cool down or warm up target anatomic structures, in order to increase thermal safety (63). Fluids freely disperse according to gravity, and tend to be distributed to dependent parts of the cavity/body; that should be taken into account when planning the procedure (63). A thorough understanding of anatomy, particularly of the retroperitoneal spaces, is paramount for an adequate peri-renal hydrodissection. The volume of necessary injected fluid is highly variable (68); a recent study demonstrated that when the hydrodissection needle tip is placed in the perirenal space, the most effective fluid accumulation usually takes place in the retromesenteric plane (besides the anterior pararenal space and the perirenal space itself) (69). Some studies state that injecting volumes of 250–500 ml results in effective protection of colon, small bowel and lumbar muscles during renal ablation (68). The literature also reports that the instillation of around 150 ml can be enough to displace adjacent bowel loops by around 2.5 cm (64, 70), although this varies widely with site of injection, patient characteristics and operator-dependent technique. Of note, during RFA procedures, hydrodissection should be performed with dextrose solutions (dextrose/water 5%); saline solution should be avoided due to its high electric conductivity (63, 64). Historically, hydrodissection was not considered suitable for cryoablation due to the hypothesis of fluid freezing upon contact with the ice ball (increasing the risk of thermal damage) (63). However, recent literature describes thermoprotective hydrodissection during cryoablation of RCC as a safe and effective technique, without compromising the efficacy of ablation at short or mid-term follow-up (68). This same report also describes in detail a proposed technique of needle(s) positioning according to the location of renal tumor relative to the ureter and pelvi-ureteric junction (68).

Gas dissection can also be used to displace and insulate organs. Gas can be injected through low-caliber needles and dedicated syringes with a luer-lock system (63). CO2 has been the preferred choice for this technique for multiple reasons: CO2 is approximately 20 times more soluble than oxygen, quickly resorbed by vessels and eliminated by respiration; it has lower thermal conductivity than that of air and water; it has low cost, nonallergic properties and lacks renal or hepatic toxicity (63, 64). Gas motion respects gravity and tends to be distributed to non-dependent parts (63). The required injection volume of gas is highly variable, but due to its quick resorption, re-injection and monitoring with intermittent CT during ablation is necessary to ensure adequate thermal protection (68). The interventional radiologist should also consider that gas has no cooling or heating properties and that it is not suitable for ultrasound-guided procedures (63).

Besides fluids and gas, novel thermoprotective agents have been recently developed, including autologous blood, fibrillar collagen, hyaluronic acid gel and polymerized thermo-protective gels (68); however, currently there is not enough evidence to support their routine clinical use.

**Figure 3:**
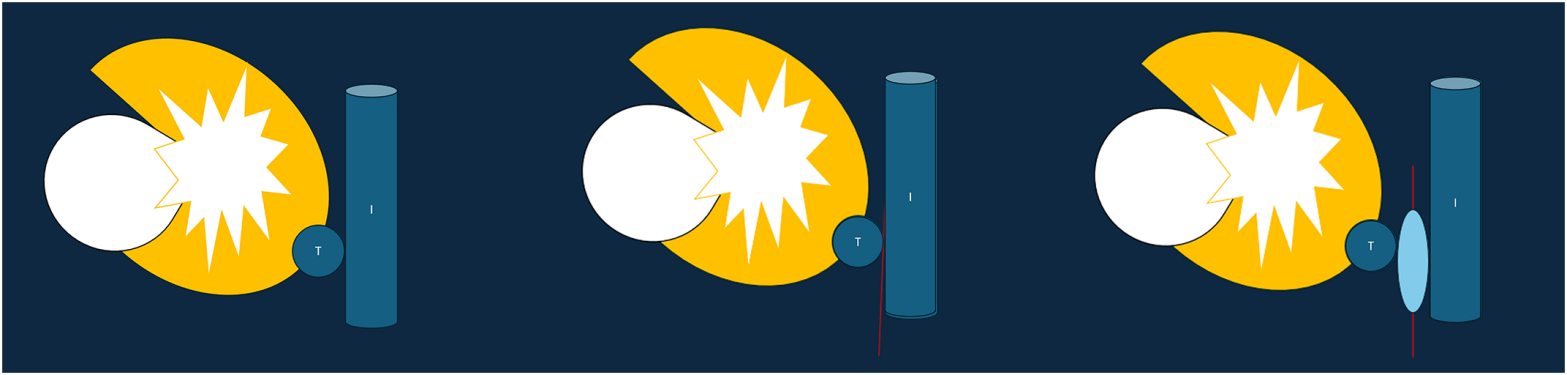
Schematic representationof baloon interposition (T = tumor, I = intestine).

Balloon interposition is usually recommended as a second-line technique, with the advantages of precise positioning and non-gravity-dependent distribution (Figure 3) (63, 64, 68). Usually, an 18–19G co-axial needle and a sheath over a 0.035-inch stiff wire are used to deploy angioplasty or esophageal balloons in target areas (between the ablation zone and the organs at risk) (63, 64, 68). Since balloon retraction is easier than advancement, some authors recommend to initially insert the sheath/balloon slightly beyond the target position (68). When the balloon is in its desired position, it is usually insufﬂated with air (or fluid) (63). Several balloons may be necessary (68).

Torquing refers to manual traction and leveraging of probes to physically displace target lesions away from vulnerable organs. This technique has been mainly used with expandable RFA devices (68), with the tines anchoring the probe inside the lesion and allowing for its mobilization and angulation; and also torquing with cryoablation, where cryoadhesion is possible with lower freezing power/“stick mode” of the cryoprobe, enabling lesion mobility before completing the cryoablation (68). Torquing has been described in many organs, but is particularly useful in lung ablation, where great tumor retraction is possible due to lung tissue compliance (68). This technique can also be used during renal ablation, but with limited efficacy (68), probably due to renal tissue characteristics and retroperitoneal location, and should be used with caution.

### 3.3 Pyeloperfusion

**Figure 4:**
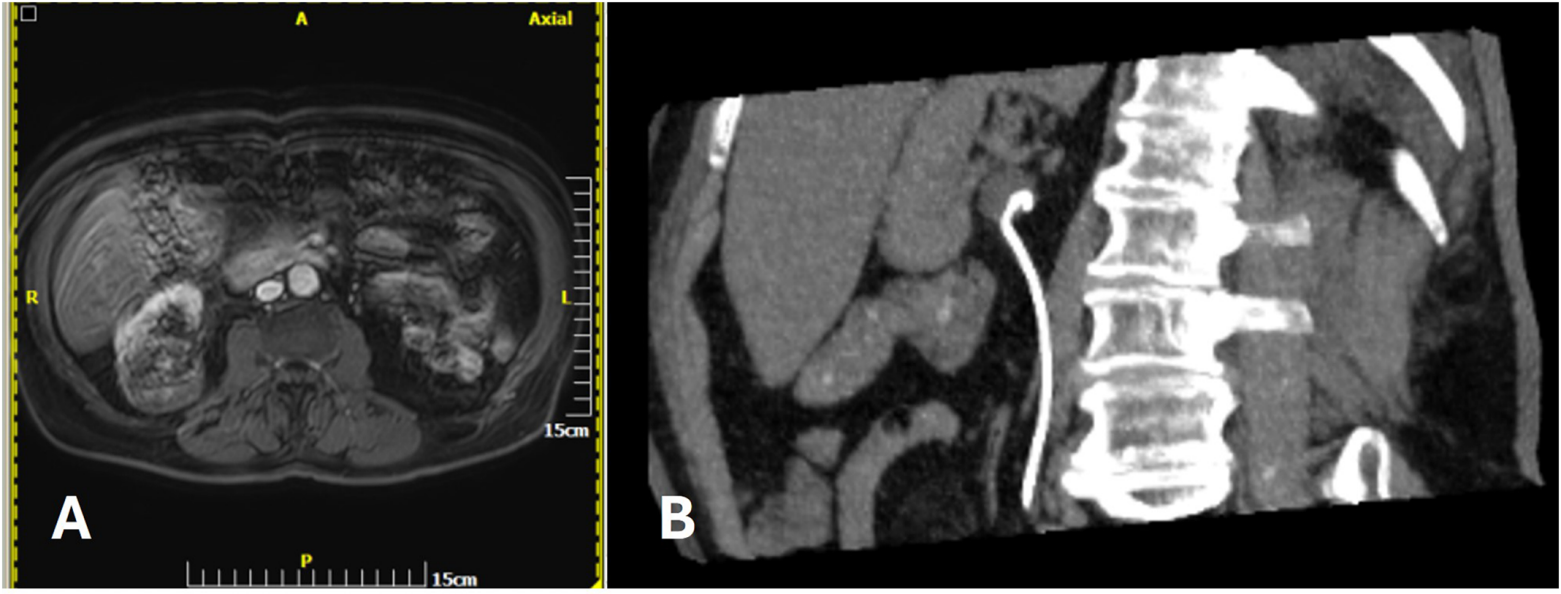
**(A)** A 74 y.o. female patient with a centrally ocated biopsy proven RCC. **(B)** The morning of percutaneous MWA a JJ stent was endoscopically plced.

Continuous perfusion of the collecting system is used to limit the risk of thermal damage (which may lead to ureteral strictures or urinoma), signiﬁcantly decreasing the number of complications (68). This technique is of particular importance during ablation of renal tumors adjacent to the ureter and pelvic-ureteric junction. Antegrade pyeloperfusion requires a percutaneous nephrostomy tube, and retrograde pyeloperfusion requires a single J catheter endoscopically positioned in the renal pelvis (Figure 4) (64, 68).

In both approaches, a Foley catheter in the bladder is required to drain the injected fluid (68). Cooled (2–6°C) or warmed (38–40°C) fluids may be used for heat-based ablation and cryoablation procedures, respectively (34, 68). There is no defined recommended ﬂow rate of injection in the literature; however, for retrograde perfusion, a pressure of 80 cm H20 for a total volume of up to 2l has been recommended (70). Currently there are no studies comparing anterograde and retrograde approaches, but retrograde pyeloperfusion is mostly favored in the literature (possibly due to easier anatomical access and less risk of complications) (68).

### 3.4 Skin protection

**Figure 5:**
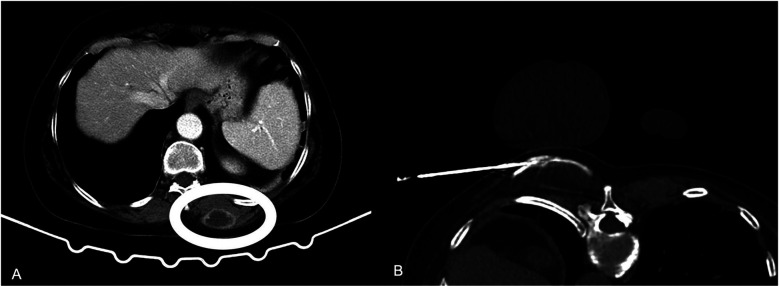
**(A)** A 64 y.o. female patient with an unresectable left kidney sarcoma a painful soft tissue metastasis of left paraspinal muscles (white circle). **(B)** A hydrodissection needle was pleced in the subcutaneous tissue for injection of local anesthetic diluted in normal saline and mixed with contrast medium. In addition during the freezing cycles a sterile glove with warm saline was placed over the skin to avoid frostbites.

In order to protect the skin from possible “frost-bite” lesions during renal cryoablation (due to the freezing of cryoprobes), a sterile surgical glove ﬁlled with warm water or saline may be placed superficially (Figure 5) (63),. When it comes to the ablation of very superficial lesions, subdermal injection of fluid or 10 ml of lidocaine 1% is an effective strategy for thermal protection of the skin (37), although this is rarely necessary in the setting of renal ablation due to the usual topography of target lesions.

## 4 Future directions

As percutaneous ablation of RCC is increasingly regarded as a safe and effective technique for the treatment of T1a tumors (14, 34), but also an option for T1b renal masses (14), the need for high quality data also increases; more specifically, in the setting of larger renal tumors, where adjunctive techniques (like pre-ablation TAE and thermal protection measures) may have a more prominent role, some issues need to be addressed: a more defined and homogeneous criteria for patient selection who will require pre-ablation TAE, the best time interval between TAE and ablation, and also the best choice of embolic material and technique. In this context, a multi-center, single-arm, prospective trial named EMBARC (Embolization Before Ablation of Renal Cell Carcinoma) (ClinicalTrials ID: NCT05410509) is currently ongoing, to evaluate safety, feasibility, technical and clinical outcomes of percutaneous cryoablation with neo-adjuvant TAE of the renal mass in patients with T1b RCC. Further investigations should: include larger cohorts, randomized controlled trials and prospective data; expand the data on RFA, cryoablation and MWA procedures as stand-alone treatment options and in the setting of combined therapies with TAE; evaluate newer ablative technologies including IRE and HIFU.

## 5 Conclusion

Percutaneous ablation of RCC is an established safe and effective technique to treat T1a and some T1b RCCs, with lower costs, shorter duration of hospitalization and lower complication rates compared to surgical approaches. In larger renal tumors, adjunctive techniques like pre-ablation TAE may be useful to decrease the rate of complications (including bleeding), improve target visualization and improve local tumor control. Different thermal protective strategies are also important to improve the safety and outcomes of ablative procedures. Further prospective data is needed to better define the indications and outcomes of these combined therapies.
